# Childbirth simulation to assess cephalopelvic disproportion and chances for failed labor in a French population

**DOI:** 10.1038/s41598-023-28459-6

**Published:** 2023-01-20

**Authors:** Olivier Ami, Jean-Christophe Maran, Albert Cohen, Israel Hendler, Eric Zabukovek, Louis Boyer

**Affiliations:** 1 Ramsay Sante La Muette, 4 Rue de Sontay, 75116 Paris, France; 2 Service de radiologie CHU Gabriel Montpied, Clermont Ferrand University Hospital, TGI –Institut Pascal, UMR 6602 UCA/CNRS/SIGMA Clermont Ferrand, Clermont Ferrand, France; 3 Réseau d’Imagerie Paris Nord, Paris, France; 4 Clinique de l’Estrée, ELSAN, Stains, France; 5 Department of Obstetrics and Gynecology, Sheba Medical Center, Tel Hashomer Affiliated to Sackler School of Medicine, Tel-Aviv University, Tel-Aviv, Israel

**Keywords:** Computational models, Magnetic resonance imaging, Neonatal brain damage

## Abstract

Reducing failed labor and emergency cesarean section (CS) rates is an important goal. A childbirth simulation tool (PREDIBIRTH software and SIM37 platform) that evaluates a 5-min magnetic resonance imaging (MRI) assessment performed at 37 weeks of gestation was developed to enhance the consulting obstetrician’s ability to predict the optimal delivery mode. We aimed to determine the potential value of this childbirth simulation tool in facilitating the selection of an optimal delivery mode for both mother and infant. A retrospective cohort study was performed on all patients referred by their obstetricians to our level 2 maternity radiology department between December 15, 2015 and November 15, 2016, to undergo MRI pelvimetry at approximately 37 weeks of gestation. The childbirth simulation software was employed to predict the optimal delivery mode based on the assessment of cephalopelvic disproportion. The prediction was compared with the actual outcome for each case. Including childbirth simulations in the decision-making process had the potential to reduce emergency CSs, inappropriately scheduled CSs, and instrumental vaginal deliveries by up to 30.1%, 20.7%, and 20.0%, respectively. Although the use of the simulation tool might not have affected the overall CS rate, consideration of predicted birthing outcomes has the potential to improve the allocation between scheduled CS and trial of labor. The routine use of childbirth simulation software as a clinical support tool when choosing the optimal delivery mode for singleton pregnancies with a cephalic presentation could reduce the number of emergency CSs, insufficiently justified CSs, and instrumental deliveries.

## Introduction

Cephalopelvic disproportion (CPD) occurs when there is mismatch between the size and/or shape of the fetal head and size and/or shape of the maternal pelvis, resulting in "failure to progress" in labor for mechanical reasons^1^. Untreated, the consequence is obstructed labor that can increase maternal or perinatal mortality or morbidity to the mother and/or the infant in the absence of a cesarean section (CS)^2,3^.

Approximately 10% of parturients in developed countries with cephalic presentation at term have an abnormality in labor progression^4,5^. In 75–80% of cases, delivery ends by the natural route; in 20–25% of cases, however, a CS is required. Thus, dystocia is the most common indication for CS during labor, with fetal distress being the second most common. CPD-related obstructed labor also accounts for 3–8% of maternal deaths worldwide.

Many studies have demonstrated the poor relevance of radiological pelvimetry alone for predicting the mode of delivery^6,7^. Radiological pelvimetry simply states whether the pelvis is normal; it does not predict whether the child can pass through the measured pelvis without CPD^8^.

The role of fetal head compliance as a confounding factor in the ability to predict CPD from a simple radiological confrontation between the fetal head and the maternal pelvis has been demonstrated in a previous work^9,10^.

Without access to a reliable tool to predict the optimal delivery mode, obstetricians are frequently left with their experience and gut feelings as the only tools with which to support their choices regarding the most appropriate delivery modes, resulting in wide margins of error. Thus, many women who fail to deliver normally experience traumatic births, and many unnecessary scheduled CSs are likely being performed^8^.

Here, we present a retrospective study examining the ability of PREDIBIRTH™ childbirth simulation software^11–13^ (Babyprogress^®^) to detect CPD using magnetic resonance imaging (MRI) pelvimetry data collected at 37 weeks of gestational age. We also assessed the potential for this tool to prevent or reduce biomechanical risks during labor. The PREDIBIRTH™ software was accessed by Software as a Service mode through the SIM37™ platform.

Our aim was to determine the potential value of the PREDIBIRTH™ tool for predicting the optimal delivery mode based on the pre-delivery identification of CPD. The results predicted by the simulation tool were compared with the results from the actual delivery to determine the predictive accuracy of SIM37™.

## Materials and methods

We conducted a retrospective observational study by analyzing the MRI results of 401 patients from the Northern Paris Imaging Network (RIPN, *Réseau d’Imagerie Paris Nord*). These patients were referred by their obstetricians for MRI pelvimetry at approximately 37 weeks of gestation. MRI examinations have replaced pelvic computed tomography scans because they protect pregnant women from unnecessary radiation exposure, in compliance with the recommendations of the Council Directive 97/43/EURATOM, Ordinance 2001-270 of March 28, 2001.

Patient data files were obtained through an MR 003 procedure filed with the National Commission for Informatics and Freedoms (CNIL, *Commission Nationale de l’Informatique et des Libertés*) to obtain a database deposited with CNIL under number 2099722v0. All patients were informed of the anonymous use of their data. According to current French regulations, written consent to participate in an anonymous retrospective study is not required. The study was conducted in accordance with relevant guidelines and regulations.

Key inclusion criteria were patients who underwent an MRI examination between 36 and 38 weeks of gestation, with no obstetric complications, a cephalic fetal presentation, and singleton pregnancy. The exclusion criteria were nonvertex fetal presentation, multiple pregnancies, being younger than 18 years, having protected adult status, and the lack of verbal consent to allow the anonymous use of their data.

In France, labor comprises 3 stages. The first stage begins with the first regular contractions and ends when the cervix is fully dilated. It includes a latency phase that begins with the first contractions, followed by an active phase that begins at 6 cm of cervical dilation and ends at full dilation. The second stage begins at full dilation and ends with the birth of the child. Classically, there is a passive phase or descent phase (between the first vaginal touch with complete dilation and the first expulsive efforts) and an active phase or expulsion phase that begins with the expulsive efforts. The third stage begins with the birth of the child and ends with the delivery, including the expulsion of the placenta.

In this study, labor management was performed in accordance with French professional recommendations of good practice. Failure of artificial induction of labor was defined as the absence of entry into labor 18 h after the start of the protocol. Operative delivery by forceps or vacuum was performed when the expulsive efforts were superior to 30 min with a normal cardiac rhythm, or in case of fetal cardiac rhythm at risk of acidosis during expulsion on an engaged presentation. Instrumental extractions and emergency caesarean sections were considered to be related to cephalopelvic disproportion when there was a slowing of labor, stagnation of cervix dilation, or non-progression of the fetal presentation, with or without abnormality of the fetal heart rate.

The MRI procedures were performed on all patients referred to the radiology department between December 15, 2015 and November 15, 2016. The images acquired enabled the visualization of the fetus and the maternal pelvis. No patients were injected with contrast agents for this examination, and none of the study patients experienced MRI-related adverse events. The Philips Ingenia 1.5 T MRI system (Philips, Netherlands) was employed to perform a 3D T1 single-shot balanced fast field echo sequence (E-Thrive, Philips) without fat saturation using the following parameters: echo time, 1.85 ms; repetition time, 3.70 ms; slice thickness, 2.5 mm; number of signal averages, 1; matrix, 140 × 167 pixels; angle, 50°; turbo field echo factor, 82; 172 contiguous slices; size of the acquisition voxel, 2.5 × 2.5 × 2.5 mm^3^; and the size of the reconstructed voxel, 1.37 × 1.37 × 2.5 mm^3^. The sequences lasted a mean of 72 s, and the examinations lasted a maximum of 5 min.

Pelvimetry assessments were performed by the radiology team. The Magnin index, which is routinely used in France, was calculated by adding the obstetric conjugate diameter to the median transverse diameter^14^. Magnin index thresholds for observed outcomes after a vaginal birth attempt are as follows: normal, > 23 cm; favorable, > 22 cm; uncertain, 21–22 cm; poor, 20–21 cm; and very poor, < 20 cm.

A childbirth simulation was performed using PREDIBIRTH™ software^15^ (Patent Cooperation Treaty: PCT/IB2011/002573), facilitating the generation of a simulated trial of labor (STOL) score, which could potentially assist in the decision-making process. The STOL score represents the level of fetal brain compression that occurs during the childbirth simulation, expressed as a percentage of fetal head molding measured after the virtual birthing process is complete compared with the initial fetal head size and shape before entering the superior inlet of the birth canal. The maximum (greatest risk) score is 8 points.

The data were analyzed using 2 methods. First, we grouped the STOL scores into 3 categories associated with different clinical presentations : STOL scores from 1 to 3 were classified as favorable, representative of a low risk of fetal brain compression and a high probability for successful normal vaginal delivery; STOL scores from 4 to 6 were classified as neutral, representative of a medium risk of brain compression, a medium probability for successful spontaneous delivery, and an increased likelihood to require instrumental delivery; and STOL scores of 7 or 8 were classified as unfavorable, representative of the greatest brain compression risk, more often associated with cephalopelvic disproportion, and good candidates for scheduled CS. Marginal means were then calculated for this model and compared using Tukey’s honestly significant difference (HSD) test. Second, we analyzed delivery modes versus STOL scores as a continuous variable. We tested pairwise differences of slopes (the effect of STOL scores) on the probabilities of decisions by the nonparametric bootstrap of differences. In both cases, models were constructed using multinomial logistic regression with the R nnet library^16^.

The McFadden, Nagelkerke, and “count” (success) pseudo-R^2^ estimation methods were used to present different aspects of the model. McFadden’s R^2^ value attempts to compromise between expressing the variability and the improvement of the model over the null model; Nagelkerke’s R^2^ estimates how much the model improves on the null model; and the count model is a direct measure of how well the model predicts outcomes. Each pseudo-R^2^ has a range from 0 to 1.

Prediction of a successful trial of labor (TOL) between the STOL scores and the Magnin index were compared by generating receiver operating characteristic (ROC) curves by generating 2 binomial models comparing either the STOL score or Magnin index vs a “successful trial”, which we defined as any vaginal delivery vs. a “failed trial”, which was any CS except for a scheduled CS. The area under the curve (AUC) and Youden’s J index were calculated and compared, with confidence intervals of differences generated by bootstrapping logistic models and recalculating STOLs.

A non-parametric Kruskal–Wallis H test with Delivery as the dependent variable and Vcat as the grouping variable was performed to test the hypothesis that birth outcomes would be distributed differently according to STOL category.

Statistical analyses were performed using R software packages (R Core Team, 2017, Vienna, Austria; https://www.R-project.org/)^17^. The figures showing our results were created using Microsoft Excel, R package ggplot2^18^, and Visualizing Categorical Data^19^ (R package version 1.4-4)^20^.

### Ethical approval

Institutional Review Board approval was not required because it was a retrospective study.

### Informed consent

Written informed consent was not required for this study because it was a retrospective study.

## Results

Of the 401 patients referred for pelvimetry examinations (Table 1), 87 (21.7%) underwent scheduled CS procedures, and 314 (78.3%) experienced a TOL based on recommendations following physician consultation. Of the women who experienced a TOL, 183 (45.6% of the total study population and 58.3% of the TOL population) experienced a normal delivery; 53 underwent an instrumental vaginal delivery (13.2% of the total study population and 16.9% of the TOL population); and 78 (19.5% of the total study population and 24.8% of the TOL population) underwent an emergency CS. The total CS rate, including both scheduled and emergency CS procedures, was 41.1% (n = 165).

**Table 1:** Study population characteristics.

Attribute	Mean ± SD	Median + 84^th^/ -16^th^ %iles^th^
Patient age, years	31 ± 5.3	31 + 5/− 6
Fetal weight at birth, g	3371 ± 468.0	3350 + 460/− 417
Time from MRI to childbirth, days	14 ± 9.3	13 + 10/− 8
Apgar score at 1 min	10 ± 1.1	10 + 0/− 0
Apgar score at 5 min	10 ± 0.7	10 + 0/− 0
Median Transverse diameter*	12 ± 0.8	12.3 + 0.8/− 0.8
Obstetric conjugate diameter*	12 ± 1.1	11.7 + 1.1/− 1.0
Bisciatic diameter*	11 ± 1.0	10.7 + 1/− 0.9
Magnin index*	24 ± 1.4	24 + 1.3/− 1.5
Biparietal diameter*	10 ± 0.4	9.7 + 0.4/− 0.5

Based on the calculated STOL scores, 125 (31.2%) patients had an unfavorable score, 101 (25.2%) had a neutral score, and 175 (43.6%) had a favorable score.

A total of 45 (36%) patients in the unfavorable STOL group and 27 (52%) patients in the emergency CS group had normal Magnin index values greater than 23 cm (Table 2). In the scheduled CS group, 38 (44%) patients had Magnin index values less than 23 cm, and none had a favorable STOL score.

**Table 2:** STOL score compared with real delivery route.

STOL score	Delivery Route						Total (%)
	Scheduled CS (%)	Normal delivery (%)	Instrumental vaginal delivery r* (%)	Instrumental vaginal delivery u* (%)	Emergency CS r* (%)	Emergency CS u* (%)	
1–3	4.5	26.9	3.7	2.2	1.5	4.7	43.6
4–6	5.2	10.7	3.0	1.2	4.0	1.0	25.2
7–8	12.0	8.0	2.5	0.5	7.5	0.7	31.2
Total	21.7	45.6	9.2	4.0	13.0	6.5	100.0

A Kruskal–Wallis H test showed that there was a statistically significant difference in delivery route between the different STOL categories, H = 6.136, p = 0.047. *CS* cesarean section, r* related to cephalopelvic disproportion, u* unrelated to cephalopelvic disproportion, *STOL* simulated trial of labour.

In the scheduled CS group, 47% were scheduled for CS due to uterine scarring, 30% were scheduled due to a history of traumatic childbirth, and 23% were scheduled due to other causes. The proportion of patients with a Magnin index less than 22 was 19% in the emergency CS group, 1% in the normal delivery group, and 5% in the instrumental vaginal delivery group.

Considering the entire study population, a total of 183 (45.6%) patients gave birth normally, 108 (59.0%) of whom had a STOL indicating a favorable outcome.

A total of 48 (55.2%) patients scheduled for CS had an unfavorable STOL score, which predicted cephalopelvic disproportion and reinforced the observed relevant indications for these cases, whereas 18 (20.7%) had a favorable STOL score, and 21 (24.3%) patients had a neutral STOL score.

**Figure 1:**
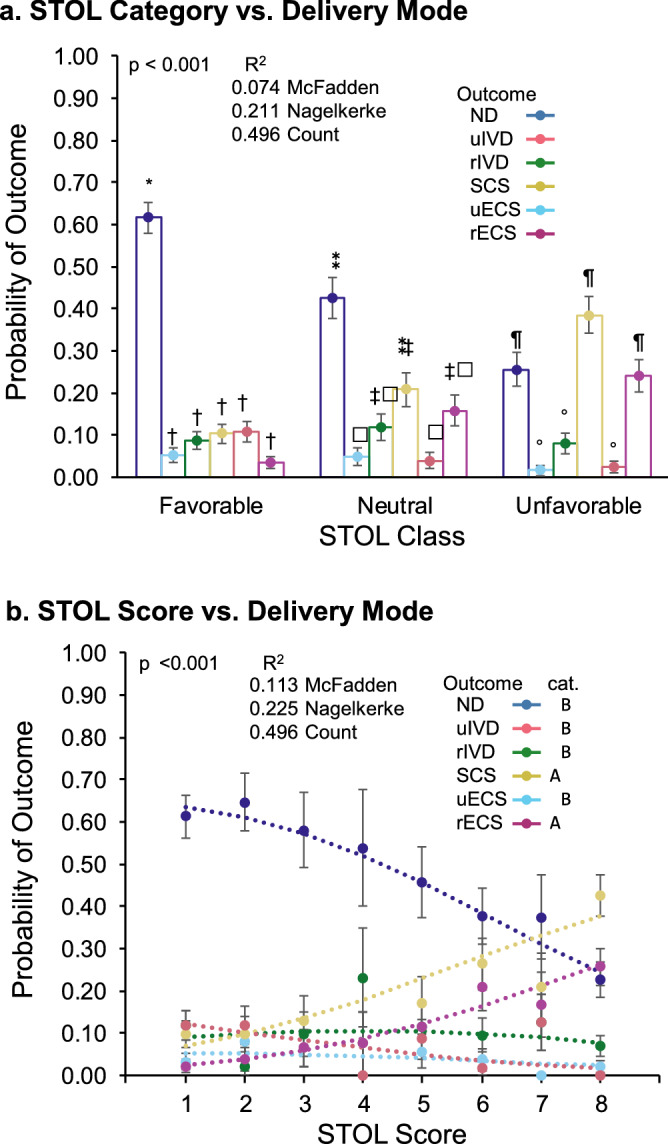
STOL models versus delivery modes. We modeled the STOL categories (favorable, neutral, unfavorable) and the STOL scores against various birth outcomes using multinomial logistic models. R^2^ values are adjusted McFadden pseudo-R^2^ values. (**a**) STOL categories vs. delivery modes. Columns represent probabilities of each outcome vs. indicated STOL category. Error bars are standard errors calculated for ratios/probabilities. The symbols above each column indicate significant differences among the probabilities within each category (marginal means). Two outcomes that share a symbol do not differ significantly within that category. Specifically: favorable has 2 groups (*, †); neutral has 3 overlapping groups (⁑, ‡, δ); and unfavorable has 2 groups (¶, °). Comparisons were not performed between categories. *CS* Cesarean Section, r* related to cephalopelvic disproportion, u* unrelated to cephalopelvic disproportion, *STOL* simulated trial of labor. (**b**) STOL scores versus delivery mode probabilities. Lines represent predicted probabilities of outcomes, treating STOL as a continuous variable. Models of logistic, linear predictor slopes were compared by bootstraps of pairwise differences. The probabilities of scheduled CS and emergency CS procedures related to cephalopelvic disproportion (labeled **a**) increased significantly with increasing STOL scores compared with all other outcomes (labeled **b**). Slopes were transformed from the model (logit) to the response scale (probability) for the figure. Points are measured as outcome probabilities, and error bars are standard errors for ratios/probabilities. *CS* cesarean section, r* related to cephalopelvic disproportion, u* unrelated to cephalopelvic disproportion, *STOL* simulated trial of labor.

We performed multinomial logistic regression, followed by the evaluation of estimated marginal means, to test potential relationships between STOL categories (favorable, neutral, and unfavorable) and the actual delivery outcomes (Fig. 1a). The analysis of variance for the model showed a significant effect of the STOL categories: Χ_2_(10) = 89.512; p < 0.001. When testing the marginal means, using Tukey’s HSD test adjusted for p ≤ 0.05, we found that the significantly most common outcome observed among those with a STOL classification of favorable was a normal delivery, whereas no significant differences in frequency were observed between the other outcomes among those with a favorable STOL classification. The unfavorable category could also be divided into 2 groups: a group with higher probabilities consisting of normal delivery, scheduled CS, and emergency CS related to cephalopelvic disproportion; and a second group comprised of the remaining outcomes. Finally, the neutral category had a more complex relationship with the outcomes. The overall trends appeared to show an inverse association between STOL values and normal delivery, with a direct association between STOL values, scheduled CS, and emergency CS related to cephalopelvic disproportion. Thus, an increased STOL score was associated with a decreased normal delivery rate and an increased rate of scheduled CS and emergency CS related to cephalopelvic disproportion.

To explicitly test these relationships, we also modeled the outcomes compared with STOL scores as a continuous variable (Fig. 1b). The predicted probabilities calculated for each STOL score value between 1 (higher proportions of low-risk deliveries) and 8 (lower proportions of low-risk deliveries) illustrated the changes in outcome probabilities associated with increasing STOL scores. This model allowed us to compare the slopes of the outcome probabilities against increasing STOL scores. Bootstrap testing of pairwise differences revealed 2 groups. As the STOL score increased, the probability of experiencing either a scheduled CS or an emergency CS related to cephalopelvic disproportion increased significantly. The remaining group was comprised of all other outcomes, which showed reduced probabilities with increasing STOL score.

Following standard PREDIBIRTH recommendations, with no other considerations (score 7–8 indicates a CS could be scheduled), would have resulted in 276 TOLs, including 45 patients who had a final outcome of emergency CS procedures as the actual outcome (11.2% of the total study population and 16.3% of the TOL population). Therefore, the number of total emergency CS procedures and emergency CSs related to cephalopelvic disproportion could potentially have been reduced by 33 (out of 78) and 30 (out of 52), respectively (57.7% and 42.3%, respectively). Furthermore, following the PREDIBIRTH recommendations alone would have resulted in 170 patients undergoing CS procedures (42.4% of the total study population), including 125 scheduled CS procedures (31.2% of the total study population) and 45 emergency CS procedures. This predicted CS proportion of 42.4% of total outcomes differed very little from the actual value of 41.1% (126 versus 165). However, 11.2% of the total deliveries would have occurred due to emergency CS (26.5% of total CS), whereas 21.2% of total deliveries would have occurred by scheduled CS (73.5% of total CS), compared with the actual emergency CS and scheduled CS rates of 19.5% (47.2% of total CS) and 21.7% (52.7% of total CS), respectively. A full sensitivity analysis for every PREDIBIRTH cutoff (Table 3) revealed the predicted outcomes had PREDIBIRTH been applied at the least (score = 1, all patients would be scheduled for CS) to most (score = 8) stringent criteria.

**Table 3:** Sensitivity analysis of PREDIBIRTH score cutoffs vs. outcomes.

	Observed	STOL threshold							
		1	2	3	4	5	6	7	8
Events									
Trials of labor	314	0	93	144	175	188	223	276	300
Emergency CS, all	78	0	13	21	25	26	33	45	52
Emergency CS, r*	52	0	2	4	6	7	11	22	26
Reduction, emergency CS	0	78	65	57	53	52	45	33	26
Reduction, emergency CS r*	0	52	50	48	46	45	41	30	26
Total CS	165	401	321	278	251	239	211	170	153
Scheduled CS	87	401	308	257	226	213	178	125	101
Percent events									
Emergency CS/all deliveries	19.5%	0.0%	3.2%	5.2%	6.2%	6.5%	8.2%	11.2%	13.0%
Emergency CS/trial of labor	24.8%	NA	14.0%	14.6%	14.3%	13.8%	14.8%	16.3%	17.3%
Emergency CS/all CS	47.3%	0.0%	4.0%	7.6%	10.0%	10.9%	15.6%	26.5%	34.0%
% reduction emergency CS	0.0%	100.0%	83.3%	73.1%	67.9%	66.7%	57.7%	42.3%	33.3%
% reduction emergency CS r*	0.0%	100.0%	96.2%	92.3%	88.5%	86.5%	78.8%	57.7%	50.0%
All CS/all deliveries	41.1%	100.0%	80.0%	69.3%	62.6%	59.6%	52.6%	42.4%	38.2%
Scheduled CS/all deliveries	21.7%	100.0%	76.8%	64.1%	56.4%	53.1%	44.4%	31.2%	25.2%

*CS* cesarean section, r* related to cephalopelvic disproportion, *STOL* simulated trial of labor.

**Figure 2:**
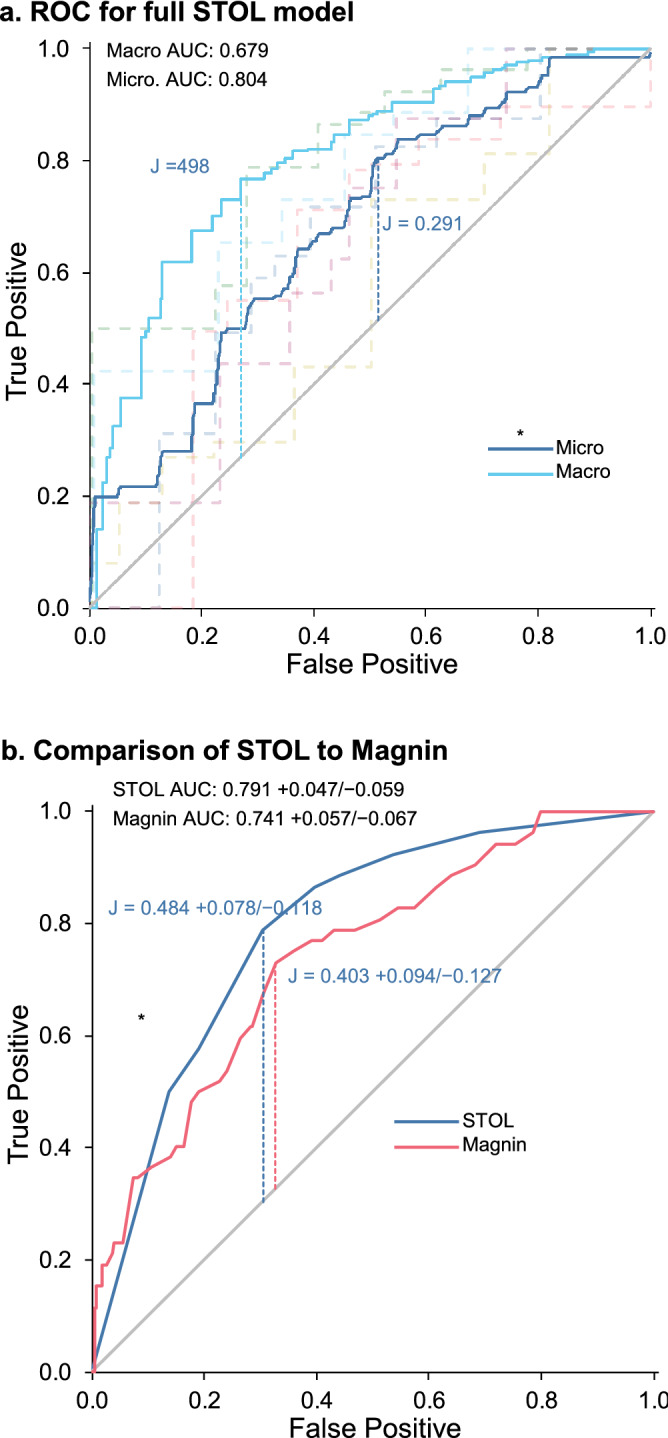
ROC analysis of STOL vs each and all outcomes, and ROC analysis comparing successful trial of labor vs CS for STOL and for Magnin scores. (**a**) ROC corresponding to multinomial logistic model of STOL vs each outcome (Fig. 1b). Both micro and macro mean AUCs are calculated, as are Youden’s J for each. Confidence intervals could not be calculated due to the bootsrap of the highly unbalanced data set not being stable. A larger sample size could compensate. (**b**) Comparison of STOL vs. Magnin in predicting CS outcome (excluding scheduled CS) vs successful trial of labor. Three methods of evaluating curve strength (AUC, Youden’s J, and Matthews correlation coefficient [MCC]) determined STOL to be superior to Magnin, as described in the text. The 95% CIs are from a nonparametric bootstrap of each model, followed by calculation of differences of AUC, J, and MCC.

An ROC analysis of the multinomial model revealed great inconsistency among different outcomes (Fig. 2a). Two averaging methods were used to estimate “overall” ROCs for the multinomial outcomes. The “macro” average gives equal weighting to each outcome. The “micro” average is based on the frequencies of each outcome. When a data set is severely unbalanced, the macro method is a preferable estimate of the overall model’s effectiveness, given that the micro method might excessively reflect input of the most common outcomes.

When we divided outcomes into successful TOL (any vaginal delivery) vs unsuccessful (any non-vaginal delivery except for scheduled CS) and we further excluded outcomes that were not related to cephalopelvic disproportion and compared ROCs for STOL vs Magnin scores (Fig. 2b), we found that the AUC for STOL was higher than for Magnin (0.791 vs. 0.740). To further explore the potential predictive superiority of STOL over Magnin, we also calculated the Matthews correlation coefficient, known as the *φ* coefficient. This measures the quality of a model and can be interpreted as a correlation coefficient. The *φ* for STOL was 0.389 and for Magnin 0.325. All 3 measures indicated some superiority for STOL over Magnin. However, we *explicitly* tested the difference between AUC, J, and *φ* values by bootstrap of the data sets, followed by modeling and calculating bootstrap ROC curves and associated statistics. We found that the difference was significant for AUC (0.050 + 0.050/− 0.043 CI) and for *φ* (0.064 + 0.107/− 0.055 CI) but not for J (0.080 + 0.092/− 0.083 CI). Thus, whether STOL is an overall better predictive method than Magnin is ambiguous.

We further explored this similarity by comparing likelihood-based entropy scores, specifically the second-order Akaike information criterion (AICc) for 4 models: STOL alone; Magnin alone; STOL + Magnin; and STOL + Magnin + their interaction. We found that, whereas the STOL alone model was estimated to be 53.3% probable (from among the models analyzed) to be the “true” model, the STOL + Magnin model had a 34.1% probability, and the model with the interaction was 12.5% probable (Table 4). Although STOL and Magnin did closely track each other (correlation coefficient =  − 742), the overlap was imperfect.

**Table 4:** Comparison of probability of STOL and Magnin models vs. successful trial of labor.

Formula	AICc	Probability (%)
STOL	220.7	53.3
STOL + Magnin	221.6	34.1
STOL + Magnin + STOL × Magnin	223.6	12.5
Magnin	234.7	0.0

In summary, the systematic application of PREDIBIRTH to our retrospective cohort would likely have resulted in a comparable overall CS rate; however, rather than an emergency CS rate of 47.2% of total CS procedures and a scheduled CS rate of 52.7% of total CS procedures, the rates would have been 30.1% and 69.8%, respectively, resulting in potentially fewer emergency CS procedures (54 versus 78) but more scheduled CS procedures (125 versus 87).

## Discussion

Currently, the main application of childbirth simulation is in the training of medical professionals^21,22^. To the knowledge of the authors, this software represents a novel 3D simulation tool that can be used to detect cephalopelvic disproportion and support clinical decision-making regarding the optimal route of delivery. The use of such tools could reduce the number of unnecessary scheduled CS procedures, emergency CS procedures, and traumatic childbirths.

The STOL score generated by PREDIBIRTH is simple to use and can assist in the classification of patients into 3 delivery categories: favorable with a high probability of a low-risk delivery (scores of 1, 2, or 3); neutral, requiring a physician to be in attendance and at an increased risk for instrumental delivery (scores of 4, 5, or 6); and unfavorable, with the potential for complications associated with cephalopelvic disproportion or likely brain compression (scores of 7 or 8), indicating that a scheduled CS could be a preferable alternative to attempted vaginal delivery.

Many of the patients in the emergency CS group were found to have cephalopelvic disproportion, and the PREDIBIRTH test is able to predict the presence of a smaller pelvis or cephalopelvic disproportion. However, the PREDIBIRTH test also demonstrated that pelvic size is not the only criterion that determines the occurrence of cephalopelvic disproportion, and only simulating the movement of the fetal head into the maternal pelvis using 3D simulation software allows for a more accurate assessment of the risks associated with childbirth.

The vast majority of traumatic deliveries in obstetrics are related to cephalopelvic disproportion, which can result in increased postpartum bleeding^23,24^, urinary incontinence^25^, fecal incontinence^26^, pelvic prolapse^27^, obstetric fistulas^28^, risk to the infant’s brain^29^, and risk to the mother’s perineum^30^. Cephalopelvic disproportion can result in a negative and traumatic experience for families, associated with difficulties in bonding between the mother and child during the first hours of the newborn’s life. When an emergency occurs during labor that subsequently leads to an instrumental vaginal delivery or emergency CS, the delivery might be perceived as a failure, with significant trauma inflicted upon the mothers^31^ and their families.

Emergency CS deliveries are associated with morbidity and mortality rates up to 7 times higher than those associated with CS deliveries that are scheduled prior to labor^32–35^, demonstrating the critical need for the early classification of high-risk patients. The ability to detect the risk of cephalopelvic disproportion and prevent traumatic labor experiences also has the potential to optimize the organization of obstetric services and patient flow management. Based on our analysis and considering the distribution of vaginal delivery failures observed in our population, use of the STOL score has the potential to enable obstetricians to select more appropriate patients for scheduled CS procedures.

Instrumental vaginal deliveries could not be definitively predicted by the application of the STOL score; however, the possibility of an instrumental vaginal delivery could be determined using the PREDIBIRTH test. Instrumental vaginal deliveries in the upper inlet are no longer recommended in most countries^36^. However, instrumental vaginal deliveries performed when the infant’s head is low enough can expedite the second phase of labor, accelerating the birth to a greater degree than the performance of an emergency CS. Therefore, the presence of few unfavorable STOL scores in the instrumental vaginal delivery group was unsurprising.

During excessively long labors, in which the infant’s brain is placed at significant risk, forceps can help to protect the brain while extracting the infant more quickly during the second phase of labor^37^. However, instrumental extractions are also associated with greater risks of maternal perineal trauma^38^.

Another concern is the perception of cephalopelvic disproportion that can have a considerable influence on the choices made by both parents and physicians^39,40^. Although the best delivery options can be obvious when dystocia is obvious, the poor outcomes associated with some TOLs require that we inform our patients of possible delivery issues, especially when they ask targeted questions. Although sometimes remaining silent might lead to better outcomes, ignoring such questions might be difficult. Problems often arise in borderline situations and are less common in more obvious situations; therefore, obstetricians are providing advice on a daily basis that is based on fuzzy logic and very personalized experience. Fortunately, decisions regarding the safest route of delivery are always made according to a set of medical arguments, following a discussion between the doctor and the patient, rather than relying on a simple imaging exam. Therefore, physicians must be able to choose from all available evidence at their disposal to justify any therapeutic proposals. However, our increasing obligation to provide information requires us to reduce the margins of error to answer our patients’ questions with increasing precision. This information is medical and should, of course, be delivered by a professional rather than a computer, and the physician and patient can have a rational discussion regarding delivery options to select the best choice for the mother and child. The use of PREDIBIRTH software, which in vast majority of cases found no brain compression in this series of patients referred by their obstetrician for pelvimetry, can help to calm fears associated with cephalopelvic disproportion for both patients and physicians and help physicians guide patients toward potentially safer options.

Future versions of PREDIBIRTH software will include recent observations regarding changes to the maternal urogenital sinus during the second phase of labor, as measured by MRI^41^, and will evaluate the stretching of the levator ani when the fetal head molding has been intense by incorporating MRI data obtained during labor. Comparison with earlier methods could be one fruitful avenue for revision. For example, based on our comparisons ranked by AICc, STOL could potentially be refined if the combination of STOL components vs obstetric conjugate diameter and median transverse diameter (Magnin components) revealed the possibility of an unaccounted-for latent variable that, if included in a revised STOL, could improve model performance.

Over the past 2 decades of use, evidence supports that performing an MRI at 37 weeks of pregnancy results in less radiation exposure than X-rays and is safe for both the mother and the fetus^42^. Having no requirement for a contrast agent is also an asset when employing this technology, avoiding the risks associated with both maternal allergy and fetal teratogenicity^43^.

This study has some limitations. It was performed as a retrospective, population-based study examining pelvimetry indications at the request of practitioners. Therefore, this patient group is likely biased toward a higher rate of significant cephalopelvic disproportion risk, which explains the high observed CS rate. This population is, therefore, not representative of the general population of France, where the official national CS rate is approximately 21%, compared with the 41.1% rate observed in our cohort.

In addition, we cannot determine what outcome would have occurred among those who underwent scheduled CS if the scheduled CS had not been performed. However, we believe that the STOL score provides useful information to determine whether women scheduled for CS based solely on the criterion of pelvis size, might otherwise successfully undergo a TOL and improve the assessment of their actual need for a CS procedure.

Prospective studies that include larger, more diverse populations remain necessary to clarify the real impacts of PREDIBIRTH as a decision support tool for delivery management guidance after 37 weeks of gestation.

## Conclusion

A simple 5-min MRI performed at 37 weeks of pregnancy and analyzed by the PREDIBIRTH childbirth simulation software tool provided useful information that could be used to determine whether women scheduled for CS based solely on the criterion of pelvis size might successfully undergo a TOL and determine their actual need for such a procedure.

Future prospective studies in this field remain necessary to ensure that future births occur under the best and safest possible conditions for both the mother and child. Such studies would require a larger and more diverse population set to ascertain the potential usefulness of this tool for applications in obstetric decision-making.

Anonymous data are available, attached to this article as the "data.xlsx" file.

## Supplementary Information

Supplementary Information.

## Abbreviations

3D: Three-dimensional
CS: Cesarean section
HSD: Honestly significant difference
MRI: Magnetic resonance imaging
STOL: Simulated trial of labor
TOL: Trial of labor
